# The role of gamma oscillations in central nervous system diseases: Mechanism and treatment

**DOI:** 10.3389/fncel.2022.962957

**Published:** 2022-07-29

**Authors:** Ao Guan, Shaoshuang Wang, Ailing Huang, Chenyue Qiu, Yansong Li, Xuying Li, Jinfei Wang, Qiang Wang, Bin Deng

**Affiliations:** ^1^Department of Anesthesiology, Center for Brain Science, The First Affiliated Hospital of Xi’an Jiaotong University, Xi’an, China; ^2^School of Medicine, Xiamen University, Xiamen, China; ^3^Department of Anesthesiology, School of Medicine, Xiang’an Hospital of Xiamen University, Xiamen University, Xiamen, China

**Keywords:** gamma oscillations, gamma entrainment, neurological function, memory, GENUS

## Abstract

Gamma oscillation is the synchronization with a frequency of 30–90 Hz of neural oscillations, which are rhythmic electric processes of neuron groups in the brain. The inhibitory interneuron network is necessary for the production of gamma oscillations, but certain disruptions such as brain inflammation, oxidative stress, and metabolic imbalances can cause this network to malfunction. Gamma oscillations specifically control the connectivity between different brain regions, which is crucial for perception, movement, memory, and emotion. Studies have linked abnormal gamma oscillations to conditions of the central nervous system, including Alzheimer’s disease, Parkinson’s disease, and schizophrenia. Evidence suggests that gamma entrainment using sensory stimuli (GENUS) provides significant neuroprotection. This review discusses the function of gamma oscillations in advanced brain activities from both a physiological and pathological standpoint, and it emphasizes gamma entrainment as a potential therapeutic approach for a range of neuropsychiatric diseases.

## Generation of gamma oscillations

Neural oscillations are rhythmic fluctuations generated by the activity of local neuron populations or neuron assemblies across brain areas and can be detected by local field potential (LFP), electrocorticography (ECoG), electroencephalography (EEG), and magnetoencephalography (MEG) at frequencies including delta (1-4 Hz), theta (4–8 Hz), alpha (8–12 Hz), beta (15–30 Hz), gamma (30–90 Hz) and high gamma(>50 Hz; Mathalon and Sohal, 2015; Cole and Voytek, 2017). They govern the timing of neuronal spikes at the microscale, and at the macroscale, they coordinate the dispersed cortical communication to enable temporal and spatial brain connectivity (Zhang et al., 2018). According to Pascal Fries’s hypothesis of “communication through coherence (CTC),” effective synaptic communication is dependent on the coordination between presynaptic and postsynaptic groups (Fries, 2015). Gamma oscillations, characterized as a rapid rhythm, allow excitation in the network to temporarily escape from the following inhibition, so enhancing the effectiveness, precision and selectivity of communication between multiple regions (Tiesinga et al., 2004). Electrophysiological data from macaques showed that virtually induced gamma synchronization between primary visual cortex (V1) and higher visual cortex (V4) facilitated sensory transmission to motor responses and shortened their reaction time, hence supporting the CTC hypothesis (Rohenkohl et al., 2018).

The discovery of three different types of narrowband gamma rhythm in primary visual cortex (V1), first reported by Han et al., advances the understanding of gamma oscillations information processing. These three gamma rhythms, referred as low gamma (25 to 40 Hz), medium gamma (40 to 65 Hz) and high gamma (65 to 85 Hz), process distinct spatial frequency signals and therefore carry selective aspects of visual information responding to the original stimulus, yet they are actually generated from different neural circuitries (Han et al., 2021). In the issue of multisensory cross-talk, it is widely assumed that the cross-modal matching of sensory signals depends on direct interaction across sensory cortices. Studies of human EEG under gamma (40 Hz) transcranial alternating current stimulation (tACS) further confirmed that corticocortical synchronized gamma oscillations implement to modulate the multisensory communication during a visual–tactile stimuli matching task (Misselhorn et al., 2019).

**FIGURE 1:**
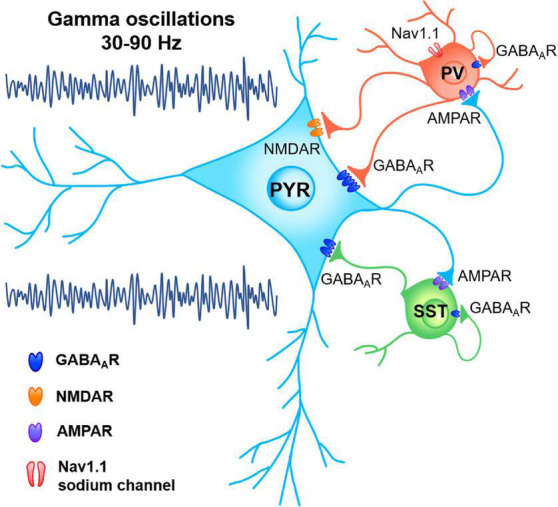
Network mechanism of gamma oscillations. Functional connectivity between pyramidal neurons (PYR) and inhibitory interneurons, including parvalbumin expressing (PV) and somatostatin expressing (SST) neurons, dynamically organized the synchronous oscillations in gamma band through pyramidal-interneuron network gamma (PING) or interneuron network gamma (ING) mechanisms.

The synchronous network oscillations in gamma band can be generated through pyramidal-interneuron network gamma (PING) or interneuron network gamma (ING) mechanisms (Whittington et al., 2000). Gamma power and frequency are modulated by the features of stimulus (direction, speed, contrast, etc.). Meanwhile, the properties of a brain, such as the resting level of GABA and size of cortex, also have an impact (Ray and Maunsell, 2015). Sensory stimulations and behavioral responses dynamically modulate the functional connectivity between pyramidal cells (PCs) and inhibitory interneurons, including parvalbumin expressing (PV +), somatostatin expressing (SST +) and vasoactive intestinal peptide (VIP) interneurons, so as to take control of the real-time interaction within the local circuits in a flexible way (Dipoppa et al., 2018; Antonoudiou et al., 2020; Figure 1). Extensive synaptic connections exist between inhibitory interneurons and their peripheral excitatory neurons, which mediates the short-term plasticity of synaptic inhibition and the consequent endogenous gamma oscillations. The prevailing understanding is that the inhibitory interneuron network characterized by these unique discharges and synaptic activities is essential for the generation of gamma rhythm (Buzsáki and Wang, 2012; Cardin, 2018). Previous studies showed the essential role of PV + and SST + interneurons to sustain gamma activity in cortex and hippocampus (HPC). The frequency of gamma oscillations is determined primarily by GABA_*A*_ receptor-induced inhibitory postsynaptic currents (IPSCs; Buzsáki and Wang, 2012; Takada et al., 2014; Pelkey et al., 2017; Antonoudiou et al., 2020). In ING network, interneurons are mutually inhibited via GABA_*A*_ receptors to quickly actualize zero-phase synchrony. During the PING mechanism, pyramidal cells elicit rapid excitation to interneurons via AMPA receptor, which consequently provides inhibition via GABA_*A*_ receptors and triggers gamma-frequency oscillations (Tiesinga and Sejnowski, 2009; Buzsáki and Wang, 2012).

Recent studies of neural computation have investigated the population dynamics of circuits composed of regularly spiking PCs, fast-spiking PV + and low-threshold spiking SST + neurons, identifying 18 potential motifs organized by diverse connection patterns between the cell types and varied input settings for the SST + cells. In addition to the classic PING and ING gamma oscillations models, the three-cell-type motifs can generate theta-nested PING and beta-nested ING gamma oscillations (Ter Wal and Tiesinga, 2021). Moreover, the dynamic shift of oscillatory pattern in V1 following visual stimulation, from gamma to beta band, is the result of competition between the projective strengths of PV + and SST + interneurons (Domhof and Tiesinga, 2021). Collectively, these findings suggest that the multi-interneuron network flexibly modulates PCs firing and consequently controls the onset and switching of brain oscillations.

In addition to GABAergic transmission, a PING network with asynchronous firing can produce gamma oscillations in response to acetylcholine (Ach) pulses via muscarinic Ach receptors (mAChRs), which enables the dynamic network adaptation during attentional tasks (Lu et al., 2020). Cue detection in behavioral attention tasks is dependent on cholinergic-driven gamma oscillations in the frontal cortex, in which both mAChRs and nicotinic Ach receptors (nAChRs) make a distinct contribution (Howe et al., 2017). Besides, NMDA receptors in PV + cells that generate relatively slow postsynaptic current are implicated in the control of spontaneous and induced gamma oscillations and have been shown to be the targets for NMDAR blockers (ketamine, MK-801, PCP, etc.) to modulate gamma oscillations (Carlén et al., 2012; Jadi et al., 2016; Bianciardi and Uhlhaas, 2021). When external stimuli drive the network into a gamma synchronic state, functional NMDA receptors are recruited at the excitatory feedback synapses between CA1 PCs and PV + interneurons to improve and stabilize neuron assemblies (Cornford et al., 2019). Nav1.1, a subunit of voltage-gated sodium channels, is expressed after neddylation modification in interneurons to maintain the excitability and excitation/inhibition (E/I) balance of GABAergic interneurons (Chen W. et al., 2021). It has been observed that Nav1.1 promotes behavior-dependent gamma oscillations in hAPP-J20 mice, which may be essential for the therapeutic advantages of interneuron transplantation in cognitive disorders (Martinez-Losa et al., 2018).

## Aberrant gamma oscillations in central nervous system diseases

Gamma oscillations have been extensively studied in the cortex, HPC, olfactory bulb (OB), amygdala, striatum, and brainstem, respectively, associated with sensation (Galuske Ralf et al., 2019; Tan et al., 2019), recognition and memory (Miller et al., 2018), locomotion (Guerra et al., 2022), emotion (Headley et al., 2021) and sleep-awake control (Garcia-Rill et al., 2013). Since precise synaptic transmission, sufficient energy supply and environmental homeostasis are indispensable for the generation of gamma oscillations, they could represent a sensitive readout of neuronal network dysfunction even before the prodromal symptoms of neurodegeneration. Previous studies have emphasized variable parameters of gamma-band oscillations in different courses of physiology or pathophysiology, including frequency, power density and cross-frequency coupling (Table 1).

**TABLE 1:** Abnormal gamma oscillations in CNS diseases.

Subject	Pathological causes	Gamma oscillations dysfunction	Behavior	References
***Learning, cognition and memory disorders***				
Aging Nfkb1-/- mice	Neuroinflammation-associated cell senescence in hippocampus	Gamma power in hippocampus ↓	Spatial discrimination and memory ↓	Fielder et al., 2020
AβO-injected mouse	AβO-induced synapse-specific dysfunctions of PV and SST interneurons	Theta-gamma power in hippocampus ↓	–	Chung et al., 2020; Park et al., 2020
Human apoE4-KI C57BL/6 mice	ApoE4-induced degeneration of GABAergic interneurons	SWR-associated slow gamma power in hippocampus ↓	Learning and memory ↓	Gillespie et al., 2016
C57BL/6 mice	Tau accumulation in astrocytes of DG	Gamma power in DG ↓	Spatial memory ↓	Richetin et al., 2020
APP/PS1 mice	Aβ-induced synaptic dysfunction between GCs and MCs	Gamma power in OB ↑	Olfactory impairment preceding learning defect	Li et al., 2019
APP/PS mice 3xTg mice	Aberrant GABAergic signaling between M/T cells and interneurons	Low-frequency gamma (40–70 Hz) power in OB ↑	Olfactory impairment preceding learning defect	Chen M. et al., 2021
***Motor dysfunction***				
PD patients	Dopamine depletion	Subthalamic gamma burst rates during movement ↓	Bradykinesia	Lofredi et al., 2018
C57BL/6 mice	Localized dopamine depletion in striatum by 6-OHDA	Striatal transient high-gamma(60–100 Hz) power ↑	Movement initiation and rotation impairment	Zemel et al., 2022
6-OHDA lesioned Wistar rats	Repeated levodopa administration-induced LID	Narrow-band high-gamma(84–113 Hz) power in M1 ↑ Gamma bursts duration and amplitude ↑	Abnormal involuntary movements ↑	Güttler et al., 2021
***Negative affection and mental disorders***				
C57BL/6J MSEW mice	Altered plasticity of PV interneurons in ventral DG	Theta power and theta-gamma coupling in ventral hippocampus ↑	Anxiety and hyperactivity	Murthy et al., 2019
C57BL/6J mice	AngII-induced synaptic plasticity and E/I disturbance	High theta(6–12 Hz)-gamma coupling in PP-DG pathway ↓	Cognitive deficit and anxiety	Gao et al., 2021
Sdy mice	Dysbindin-1 mutation-induced defective mitochondrial fission	Gamma range integrated power in CA3 ↓	Cognitive impairment relative to schizophrenia	Zhao et al., 2021
C57BL/6J mice, transient stimulation of L2/3 PYRs from P7–P11	L2/3 PYR dysfunction, altered inhibitory feedback by FSs and E/I imbalance in prefrontal circuits	Task-related gamma power in adult mPFC ↓	Long-lasting impairment of short-term and working memory, recognition and social behavior	Bitzenhofer et al., 2021
NL3 R451C KI mice	Decreased excitability of FSs, neuronal firing rates and phase-coding abnormalities	Theta-low gamma (30–50 Hz) coupling in mPFC during social interaction ↓	Social novelty deficit	Cao et al., 2018
Cntnap-/- mice	Reduction of hippocampal PV interneurons and inhibitory input to CA1 PYRs	Theta-nested mid gamma(65–90 Hz) power in CA1 ↓	Spatial discrimination deficit relevant to ASD	Paterno et al., 2021

AβO, amyloid β oligomers; PV, parvalbumin; SST, somatostatin; SWR, sharp-wave ripple; DG, dentate gyrus; GCs, granule cells; MCs, mitral cells; OB, olfactory bulb; M/T cells, mitral/tufted cells; LID, levodopa-induced dyskinesia; M1, primary motor cortex; MSEW, maternal separation with early weaning; PP, perforant pathway; mPFC, medial prefrontal cortex; PYRs, pyramidal neurons; FSs, fast-spiking interneurons; ASD, autism spectrum disorders.

### Central nervous system perturbation: Inflammation and oxidative stress

Abnormal gamma oscillations concurrent with neuroinflammation imply a disruption in the microenvironment of the neuronal network of the central nervous system (CNS). In the hippocampal CA3 of aged nfkb-/- mice, a model of low-grade sterile inflammation during the aging process or “inflammaging,” the gamma frequency area power of the oscillations is reduced (Fielder et al., 2020). As a high-frequency synchronization of neuron populations, gamma oscillations are susceptible to metabolic and oxidative stress since a quick requisition for mitochondria is necessary to meet the energy requirements imposed by neuronal activities (Kann et al., 2011). Different levels of network dysfunction are induced by TLR-activated microglia, which is primarily mediated by the excessive release of reactive oxygen and nitrogen species rather than proinflammatory cytokines (Schilling et al., 2021). In hippocampal slices *in situ*, IFN-gamma-activated microglia upregulate the expression of inducible nitric oxide synthase (iNOS) and subsequently produce a large amount of NO, resulting in a decrease in the frequency of gamma-band oscillations (Ta et al., 2019). NO is an inhibitor of complex IV of the mitochondrial respiratory chain, which stimulates the formation of oxidants and exacerbates neuronal damage. With the combination stimulation of LPS and IFN-gamma, substantial loss of PV + neurons and decreased or absent gamma oscillations are detected in HPC cells (Chausse et al., 2020). Caliskan and coworkers investigated antibiotics’ effect on behaviorally-relevant networks, showing that antibiotic-induced dysbiosis of the gut microbiota leads to microglia activation and iNOS production, as well as decreased neurotrophic factor (NGF), reduced baseline synaptic transmission, and impaired cholinergic gamma oscillations in hippocampal CA3 and CA1 (Çalışkan et al., 2022).

Studies on rodents and clinical trials have demonstrated that anti-inflammatory therapy helps to restore gamma oscillations and improve cognitive performance (Assogna et al., 2020; Fielder et al., 2020). However, non-inflammatory proliferation of microglia in hippocampal sections *in situ* induced by granulocyte macrophage colony-stimulating factor (GM-CSF) infiltration can interfere chronically with gamma oscillations, independent of the production of proinflammatory mediators (Dikmen et al., 2020). These findings indicate the susceptibility of gamma oscillations to the disruption of CNS homeostasis and emphasize the involvement of homeostatic microglia, the resident immune cells for dynamic brain surveillance, in the maintenance of gamma oscillations in the context of various disorders, which may be associated with diverse functions of microglia such as immune response, ROS synthesis, and synaptic remodeling.

### Hyperalgesia

Pain is a perceptual phenomenon formed by the dynamic integration of sensory and contextual processes (cognition, emotion, motivation, etc.), and is dependent on the spatial-temporal coordination of brain oscillations of different frequency (Ploner et al., 2017). Human EEG reveals that the intensity of pain is encoded uniquely by gamma oscillations in the medial prefrontal cortex (mPFC); yet gamma oscillations are not responsible for tracking the intensity of visual, auditory, or non-nociceptive somatosensory stimulation (Nickel et al., 2017; Hu and Iannetti, 2019). In the insula of epileptic patients, notably higher gamma oscillations were detected prior to pain perception (Liberati et al., 2018). Studies in rodents suggest that gamma oscillations mediate acute pain perception and aversion, as well as hypersensitivity to chronic pain (Tan et al., 2021). Electrophysiology provides evidence that gamma-band activities in the primary somatosensory cortex (S1) is coupled with the spike firing of interneurons in S1 superficial layer contralateral to the noxious stimulus (Yue et al., 2020). Mice suffering from mechanical injury or inflammatory pain exhibit higher gamma oscillations in S1 and nociception susceptibility. The induction of gamma oscillations in S1 via optogenetic stimulation exacerbates nociceptive hypersensitivity and aversive avoidance. (Tan et al., 2019).

### Learning, cognition and memory disorders

The common perspective is that gamma oscillations in cortex and HPC constitute the fundamental network mechanism for routing accurate information flow and communication in the processes of attention, cognition and memory (Kim et al., 2016; Tamura et al., 2017; Griffiths et al., 2019). Most recent research revealed that gamma-frequency communication between entorhinal cortex and dentate gyrus exerts a crucial role in routing task-relevant clues by modulating the recruitment of hippocampal cell assemblies, including gyrus granule cells, mossy cells and CA3 pyramidal cells, whereas disrupting gamma synchronization by optogenetic perturbation of entorhinal cortex leads to learning impairments (Fernández-Ruiz et al., 2021).

Reduced rapid (alpha, beta, and gamma) and increased slow (delta and theta) rhythms are general resting-state EEG (rsEEG) metrics in patients with Alzheimer’s disease (AD; Jafari et al., 2020). Studies in humans and rodents provide corroborative evidence of detrimental links between AD neuropathology and gamma oscillations. EEG in neurodegeneration-positive patients provides a demonstration of inverted U-shape curve relationship between amyloid β (Aβ) burden and the power spectral density of gamma oscillations, reflecting the compensatory effect of the brain in the early stage and accelerated lesions after decompensation in the later stage of neurodegeneration (Gaubert et al., 2019). Before the onset of Aβ plaque deposition and cognitive impairment, 5XFAD mice have exhibited abnormalities in sharp wave ripple (SWR)-related slow gamma oscillations (10–50 Hz; Iaccarino et al., 2016). Aβ oligomer (AβO) can selectively block IPSCs produced by PV + and SST + interneurons by augmenting the initial release of GABA, hence impairing PV-PC and SST-PC interactions at the presynaptic site. This AβO-induced suppression of CA1 PC input may underpin the asynchrony of CA1 PC firing and decrease in gamma power (Chung et al., 2020). The power of hippocampal SWR-related slow gamma oscillations significantly decreases in apoE4-KI mice, which can be restored by specific elimination of apoE4 in GABAergic interneurons in forebrain, suggesting the crucial role of SWR-related slow gamma oscillations in apoE4-mediated learning and memory disorders (Knoferle et al., 2014; Gillespie et al., 2016). In addition, the accumulation of 1N3R subtype of tau, detected in the hilar astrocytes in dentate gyrus of AD patients, triggers a drastic relocation of mitochondria in soma-processes of astrocytes and abnormal ATP synthesis, and reduces gamma oscillations in dentate gyrus, leading to the spatial memory impairment (Richetin et al., 2020).

Olfactory impairment, manifested as disability to detect and recognize odors, is one of the high incidence symptoms among AD patients. Neuropathology in olfactory processing areas, including OB and entorhinal cortex, can emerge so early that even before the onset of Aβ plague and memory impairment (Murphy, 2019). Along with the aging process, Aβ deposition damages the dendritic synaptic connection between granular cells (GCs) and mitral cells (MCs), resulting in aberrant enhancement of gamma oscillations and olfactory dysfunction (Li et al., 2019). APP/PS1 and 3xTg mice aged 3–5 months (i.e., before the formation of Aβ plaque) have exhibited early olfactory impairment, as the number of olfactory sensory neurons (OSNs) and the amplitude of olfactory potential decreased, causing the loss of glutamatergic innervation from olfactory epithelium (OE) to OB and the consequent abnormalities of mitral/tufted (M/T) cells to trigger GC and other interneurons to release GABA. The power of low-frequency gamma oscillation (40–70 Hz) in OB is abnormally enhanced (Chen M. et al., 2021). Together with these findings, it is reasonable to conceive gamma oscillations as a potential biomarker of preclinical AD.

### Motor dysfunction

The basal ganglia-thalamus-cortex circuit, which shapes voluntary movements, carries information in the form of firing frequencies, especially the gamma band that exists in many network nodes. Both beta event-related desynchronization (ERD) and gamma event-related synchronization (ERS) occur in the internal globus pallidus (GPi) prior to the preparation and execution of self-initiated and internally or externally triggered movements (Tsang et al., 2012). Human ECoG shows the enhanced phase coupling between subthalamic nucleus (STN) neuronal firing and cortical gamma oscillations before fast reaction (Fischer et al., 2020). The motor dysfunction of patients with Parkinson’s disease (PD) is interpreted as the imbalance of “antikinetic” beta and “prokinetic” gamma oscillatory patterns in the basal ganglia-thalamus-cortex loop. Bradykinesia, one of the most prominent symptoms of PD, may be caused generally by the insufficient recruitment of gamma-frequency synchronized bursts during movement processing (Lofredi et al., 2018; Guerra et al., 2022). However, hyperactive gamma oscillations also disrupt motor circuit function. Dopamine depletion induces an aberrant correlation within the GABAergic spinous projection neurons (SPNs) network in the dorsal striatum, sparking off pathologically transient high-frequency gamma (60–100 Hz) oscillations during SPN activation (Zemel et al., 2022). Levodopa-induced dyskinesia (LID), as reported by Güttler et al., is associated with the constant amplification of narrow-band gamma oscillations in primary motor cortex (M1) of levodopa-treated PD patients (Güttler et al., 2021).

### Negative affection and mental disorders

During fear expression, the basolateral amygdala (BLA) is synchronized with the HPC and mPFC via theta oscillations. As the central circuit of fear memory consolidation and extinction, the BLA-HPC-mPFC loop establishes an oscillatory mode defined by varying intensities of low/high-frequency gamma oscillations and their cross-frequency coupling with theta oscillations (Stujenske et al., 2014). In the state of anxiety and vigilance, high-frequency gamma oscillations synchronize neural firing in BLA, while mPFC and BLA are strongly entrained in the high-frequency gamma band. Although high-frequency gamma oscillations have no significant impact on the firing rate of BLA neurons, they regulate affection and behavior by dynamically altering the firing rates and synchronization of neuronal assemblies (Amir et al., 2018). As a model of early life adversity, maternal separation with early weaning (MSEW) mice demonstrate susceptibility to anxiety and hyperactivity disorders. Upon entering a novel environment, the theta oscillations and theta-gamma cross-frequency coupling in their ventral HPC significantly increase. Furthermore, the loss of PV + and SST + interneurons and increased peripheral networks (PNNs) surrounding PV + neurons are observed in the ventral dentate gyrus, which may underlie the change of neural plasticity caused by early life stress (Murthy et al., 2019). Angiotensin II (ANG II) is found to disrupt the E/I balance in HPC, particularly the perforant pathway-dentate gyrus (PP-DG) theta-gamma phase-amplitude coupling (PAC), leading to the anxiety-like behaviors of mice, which presumably contributes to the anxiety vulnerability in patients with hypertension (Gao et al., 2021).

Abnormal GABAergic signaling and NMDA receptor hypofunction play a crucial role in the pathophysiology of schizophrenia (SCZ), which disturbs the E/I balance in cortical and subcortical networks and represents aberrant neural oscillations (Nakazawa and Sapkota, 2020; Bianciardi and Uhlhaas, 2021). 40 Hz auditory steady state response (ASSR) is commonly used to evaluate the ability to generate inducible gamma oscillations, and in SCZ patients, the spectrum power and/or phase locking of 40 Hz ASSR are dramatically diminished (Thuné et al., 2016). When patients were performing cognitive and complex perception tasks, the inducible gamma oscillations in their frontal cortex is abnormally weak, which is partly due to the altered activity of PV + neurons (Gonzalez-Burgos et al., 2015). The expression of dystrobrevin binding protein-1 (dysbindin-1), a potential risk gene for SCZ, is reduced in the brain specimens of SCZ patients. Neuronal firing at gamma-frequency drives the translocation of dysbindin-1 into mitochondria, where it interacts with Drp1 and its receptors and initiates the assembly of Drp1 oligomers that induce mitochondrial fission. Consequently, this molecular depletion could inhibit mitochondrial fission and impair gamma oscillations, whereas stimulating mitochondrial division helps to restore the gamma rhythm (Wang et al., 2017; Zhao et al., 2021).

Autism spectrum disorder (ASD) is a constellation of restricted behavior, social deficit, cognition and memory impairments (Lord et al., 2018). In patients with ASD, reduction in the number of interneurons, the expression of GABA receptor subunits, as well as the level of GABA, indicate an E/I imbalance associated to GABAergic transmission (Paterno et al., 2021). During the developmental time window of high relevance for formation of synaptic contacts (postnatal day 7–11), briefly activating L2/3 pyramidal neurons in mPFC using optogenetic technique results in the premature maturation of L2/3 dendrites and out-of-balance of neuronal E/I activity, ending up with the decreased gamma oscillations in mPFC and cognitive and social deficits in adulthood (Bitzenhofer et al., 2021). Neuroligins (NLs) are essential for the formation and function of synapses. NL3 R451C-KI mice exhibit lower excitability of fast-spiking interneurons and abnormal gamma oscillations in mPFC, which could be the mechanistic underpinnings of their social novelty deficit (Cao et al., 2018). Compared to non-social engagement, the gamma power in the nucleus accumbens spontaneously increases during social interaction; however, this adaptation fails to occur in stressed animals (Iturra-Mena et al., 2019). Cntnap2 KO mice, typified as an ASD phenotype, demonstrate lower pre-somatic inhibition of pyramidal neurons due to the loss of PV + interneurons in HPC, giving rise to the significant decrease of theta-nested gamma oscillations (65–90 Hz) and SWR (Paterno et al., 2021).

## Therapeutic effect of gamma entrainment in central nervous system diseases

Despite these advances above, the question of whether gamma oscillations serve a physiological role in cortical processing responding to certain stimulus remains a controversy. Ray and Maunsell argued that gamma oscillations can be conceived as a marker of relatively local and moderate interactions involving excitation and inhibition in the cortex, yet they do not contribute to any advanced cortical functions (Ray and Maunsell, 2015). Nevertheless, modulating oscillations via sensory stimulation, transcranial electrical stimulation (tES) and deep brain stimulation (DBS), termed oscillation entrainment, have provided considerable evidence that neural oscillations are the critical mechanism operating memory encoding, consolidation, consolidation and retrieval, rather than an epiphenomenon of cognition and memory (Iturra-Mena et al., 2019; Adaikkan and Tsai, 2020; Table 2).

**TABLE 2:** Effects of gamma entrainment in CNS diseases.

Subject	Method	Affected brain area	Major findings	References
***Recognition and memory disorders***				
Healthy Long Evans rats	Optogenetic stimulation of FSs in BLA	BLA	Contextual memories consolidation↑	Kanta et al., 2019
J20-APP mice	Optogenetic stimulation of MSPV neurons (40 Hz)	Hippocampus	Hippocampal theta-low gamma phase-amplitude coupling↑ Spatial memory ↑	Etter et al., 2019
5XFAD mice	Optogenetic stimulation of FSPV interneurons (40 Hz)	CA1	Aβ levels ↓ Microglial Aβ uptake ↑	Iaccarino et al., 2016
5XFAD mice	Visual stimulation (40 Hz)	VC	Aβ levels ↓ Microglial Aβ uptake ↑	Iaccarino et al., 2016
5XFAD mice	Auditory stimulation (40 Hz)	AC Hippocampus	Recognition and spatial memory ↑ Aβ levels, Tau phosphorylation ↓ Reactive astrocytes and microglia ↑	Martorell et al., 2019
5XFAD mice	Combined visual and auditory stimulation (40 Hz)	mPFC	Reactive microglia ↑ Aβ levels ↓	Martorell et al., 2019
P301S mice CK-p25 mice	Visual stimulation (40 Hz)	V1, CA1	Learning and spatial memory ↑ Neuronal loss, Microglial inflammatory response ↓ Synaptic integrity ↑	Adaikkan et al., 2019
***Motor dysfunction***				
PD patients	DBS (130 or 160 Hz)	M1, PMC, SMA, STN and CER	Motor performance ↑ Beta power ↓ Gamma power ↑	Muthuraman et al., 2020
PD patients	tACS (70 Hz)	M1	Facilitation of MEPs, LTP-like plasticity of M1↑ SICI ↓	Guerra et al., 2020
C57/BL6J stroke mice	Optogenetic stimulation of interneurons (40 Hz)	Cortex	Motor performance ↑ Spreading depolarizations, Brain swelling and lesion volume ↓ Cerebral blood flow ↑	Balbi et al., 2021
***Mental disorders***				
C57BL/6 stroke mice	Visual stimulation (40 Hz)	Cortex, amygdala	Anxiety susceptibility to stress exposure ↓ HDAC3 and Cox1 in damaged cortex, EP2 in amygdala, Microglia activation ↓	Zhu et al., 2022
NL3 R451C KI mice	Optogenetic stimulation of PV interneurons in mPFC (40 Hz nested at 8 Hz)	mPFC	Social novel preference ↑ Theta and gamma power ↑	Cao et al., 2018

MSPV neurons, medial septum parvalbumin neurons; FSs, fast-spiking interneurons; BLA, basal lateral amygdala; VC, visual cortex; AC, auditory cortex; mPFC, medial prefrontal cortex; DBS, deep brain stimulation; tACS, transcranial alternating current stimulation; M1, primary motor cortex; PMC, premotor cortex; SMA, supplementary motor area; STN, subthalamic nucleus; CER, cerebellum; MEPs, motor-evoked potentials; SICI, short-interval intracortical inhibition; HDAC3, histone deacetylases 3; Cox1, cyclooxygenase 1.

### Gamma entrainment improves learning, cognitive and memory defects

Recent studies have explored the positive effect of gamma entrainment on various types and different stages of memory process. During the consolidation of contextual memory, gamma power in BLA increases, while externally boosting gamma synchrony in BLA can strenthen the sequential memory strength. Conversely, memorial consolidation will be impeded by diminished gamma oscillations (Kanta et al., 2019). 40 Hz optogenetic stimulation of medial septal PV + neurons restores hippocampal low-frequency gamma oscillations amplitude and theta-gamma PAC in AD mice, hence aiding in the repair of spatial memory deficits (Etter et al., 2019). Given that AβO accumulation causes injury to interneuron synapses, optogenetic activation of PV + and SST + interneurons in AβO -injected mice selectively restores the decreased peak power of theta and gamma and resynchronizes the spike firing of CA1 PYRs (Chung et al., 2020; Park et al., 2020). Consistent with previously established significance of Nav1.1 in gamma oscillations, transplantation of Nav1.1-overexpressing interneurons enhances gamma oscillations and ameliorates the epileptic-like behavior and cognitive deficit of hAPP-J20 mice (Martinez-Losa et al., 2018). Strikingly, using patterned sensory stimuli to induce neural activity and gamma entrainment, termed gamma entrainment using sensory stimulus (GENUS), work from Li-Huei Cai and her colleagues has demonstrated intriguing therapeutic effects in animal models of AD. Their strategies evoking gamma oscillations in visual cortex (VC), auditory cortex (AC), HPC and mPFC via 40Hz LED flickering, auditory stimulation or audiovisual combined stimulation conduce to improve the cognitive and spatial memory deficits in AD model mice (Iaccarino et al., 2016; Adaikkan et al., 2019; Martorell et al., 2019).

Although optogenetic and sensory stimulation have demonstrated a surprising neuroprotection effect, relatively little is understood about how gamma entrainment mediates the decrease in Aβ deposition. Studies from Tsai’s team show that optogenetic gamma stimulation or GENUS activates microglia to a phagocytotic state, moderately dilates the vessel diameter, and increases the co-localization of lipoprotein receptor-related protein 1(LRP1) and Aβ, which mediates Aβ phagocytosis by the endothelium. Besides, the levels of the cleavage intermediates and endosomal processing of Aβ precursor protein (APP) in CA1 neurons are also reduced following 40 Hz stimulation, suggesting that gamma entrainment helps to alleviate Aβ burden through both diminished amyloid generation by neurons and increased amyloid endocytosis by microglia (Iaccarino et al., 2016; Martorell et al., 2019). However, recent evidence supports that optogenetic gamma stimulation can improve memory in the absence of Aβ clearance (Etter et al., 2019). Paradoxically, Wilson et al. observed that optogenetic activation of PV + neurons in the basal forebrain induced gamma entrainment in the cortex of 5xFAD mice, which instead increases the burden of Aβ_1–42_ in frontal cortex (Wilson et al., 2020). Hence, it is speculated that different methods, such as optogenetic, visual or auditory stimulation, are likely to entrain gamma oscillations within a complex neurocircuit while crossing various brain areas, despite the fact that cellular and molecular underpinnings of gamma entrainment in memory improvement remain a mystery that is vital for further understanding and application of gamma entrainment.

To date there is little agreement on whether visual stimulation can effectively induce endogenous gamma entrainment in humans (Jones et al., 2019; Duecker et al., 2021). Applying 40 Hz transcranial magnetic stimulation (tMS) on AD patients enhanced gamma-band power in the left temporal parietal cortex and improved cognitive and executive function through boosting local, remote, and dynamic integrative activities within brain regions (Liu et al., 2021). Patients with mild cognitive impairment (MCI) receiving 1-hour daily 40 Hz audiovisual stimulation for 4–8 weeks validated GENUS as a safe and tolerable treatment, while the participants demonstrated improved connectivity between posterior cingulate cortex and precuneus lobe, and decreased level of several cytokines such as TGF-α, MIP-1β, IL-5 and TNF-like weak inducer of apoptosis (TWEAK) in cerebrospinal fluid, though Aβ and tau load remained unchanged (He Q. et al., 2021). Likewise, 40 Hz tACS treatment had no significant influence on Aβ burden but a moderate decrease of p-Tau in the targeted temporal lobe (Dhaynaut et al., 2022). Using 125Hz fast-gamma magnetic stimulation (FGMS) in patients with AD and MCI, the results showed no significant amelioration in cognition and depression (Mimenza-Alvarado et al., 2021). Given the limited sample size, non-uniformed treatment settings and evaluation criteria in the clinical trials at present, further large-scale studies are warranted to confirm a stable and reliable phenotype of the therapeutic effect of gamma entrainment on human cognitive and memory disorders.

### Gamma entrainment restores the motor dysfunction

A recent study revealed that pallidotomy, one of the oldest therapies for motor impairments such as rigidity and bradykinesia, enhances the gamma oscillations in M1, which implies the effectiveness of modulating network rhythm in PD treatment (de Hemptinne et al., 2019). The advent of DBS and tACS has made a major breakthrough in the treatment of PD, though there is limited information available about their impact on oscillation entrainment, a possible therapeutic mechanism revealed recently. Recordings from the basal ganglia and cortex of PD patients showed that DBS inhibits the antikinetic beta (13–30 Hz) while reinforces the prokinetic gamma rhythm (60–90 Hz; de Hemptinne et al., 2015; Muthuraman et al., 2020). Gamma entrainment via tACS improves motor impairment in individuals with PD, and the increase of motor amplitude during tACS is interrelated to the modulation of GABAA activity in M1 (Guerra et al., 2020). Notably, aberrant enhancement of beta-gamma PAC in premotor, primary motor and somatosensory cortex is associated with the severity of clinical PD symptoms. Future research will have to clarify the influence of interventions such as DBS and dopaminergic therapy on cross-frequency coupling (Gong et al., 2021).

Post-stroke survivors have high incidence of disabilities related to self-care and mobility. Although their gamma oscillations recorded by magnetoencephalogram (MEG) at resting state remains normal, the auditory gamma entrainment is attenuated, indicating a specific defect in the reserve of gamma oscillations. Gamma oscillations reserve in the affected cerebral hemisphere demonstrates a high correlation with the rehabilitation of patients, which suggests that gamma oscillations are probably accompanied with the whole rehabilitation process of stroke (Pellegrino et al., 2019). Few studies have investigated the effect of gamma entrainment on ischemic lesions. In the acute phase after stroke, activation of inhibitory interneurons in M1 through 40 Hz stimulation reduces the incidence of spreading depolarizations (SDs) while alleviating brain edema and lesion volume, and increasing cerebral blood flow, all of which contributes to an improvement in the motor performance of post-stroke mice (Balbi et al., 2021).

### Gamma entrainment alleviates negative affections and mental disorders

Previous studies have examined gamma oscillations in the neural circuits related to emotion and social behavior, though few attempts have been made to determine the effect of gamma entrainment on mental disorders. Work from our group demonstrated that 40 Hz visual stimulation down-regulates the expression of histone deacetylase 3 (HDAC3) and cyclooxygenase-1 (COX1) in the ischemic cortex and EP2 in the amygdala, which ameliorates the susceptibility to anxiety and depression in mice exposed to post-stroke stress (Zhu et al., 2022). 40 Hz nested at 8 Hz optogenetic activation of PV + interneurons in mPFC of NL3 R451C-KI mice effectively improves the social novelty preference, while constant 40 Hz stimulation has no significant effect (Cao et al., 2018). A number of clinical trials showed that tES and tMS is presumably a therapeutic operation for ASD patients by modulating gamma oscillations to be normalized (Casanova et al., 2020; Kayarian et al., 2020). Intriguingly, light therapy for major depressive disorder (MDD), as reported by Huang et al., performs an antidepressant effect through activating the thalamic ventral lateral geniculate nucleus and intergeniculate leaflet and lateral habenula (retina-vLGN/IGL-LHb) circuit, which provides a theoretical basis for the application of sensory stimulation in depression treatment (Huang et al., 2019).

## Mechanism of gamma entrainment therapy: Neurons and glia

So far, there is limited research investigating the mechanism of therapeutic gamma entrainment at the level of cell and molecular biology. It appears that gamma entrainment exerts an overall neuroprotective impact on neurons and glial cells, especially microglia, yet the neuron-glia crosstalk underlying gamma entrainment might be rather complicated. Moreover, it is astonishing to find that gamma entrainment is capable to drive changes of gene expression and protein phosphorylation.

### Gamma entrainment offers neuroprotection

40 Hz visual stimulation significantly reduced neuronal loss in V1, CA1, somatosensory cortex and cingulate cortex of mouse models of neurodegeneration, as well as the loss of CA1 excitatory neurons in ischemic stroke mice, yet the mechanism of this effect is not well understood. Studies from Adaikkan et al. showed that GENUS down-regulates the expression of inflammatory genes and reduces DNA damage. The administration of GENUS protects P301S and CK-p25 mice from suffering severe neuronal loss and brain atrophy (Adaikkan et al., 2019). Intriguingly, the rescued neuronal survival after ischemia is independent of any change of cerebral blood flow or microglia response, implying that visual stimulation may exert a direct effect on neurons. As Zhang et al. hypothesized, enhancement of CA3-CA1 excitatory synaptic transmission under gamma entrainment sends pro-survival signals to the CA1 neurons, so making CA1 neurons more resistant to reperfusion-induced neuronal death (Zheng et al., 2020).

### Gamma entrainment modulates neuronal connections

Synchronized synaptic activity of neuronal populations are the major microevents underlying neural oscillations measured at the level of LFP. The activity-dependent accommodation of neuronal connections, also known as synaptic plasticity, is closely allied with neural oscillations (Bocchio et al., 2017).

Regulated by metabotropic glutamate receptor 5 (mGlu5), synaptic plasticity plays a pivotal role in hippocampal theta and gamma oscillations induced by high-frequency afferent stimulation. This oscillatory response not only represents the amplitude and persistence of synaptic efficiency, but is internally associated with the successful generation of hippocampal long-term potentiation (LTP; Bikbaev and Manahan-Vaughan, 2017). PV + interneurons mediate LTP for E→I synapses (LTP_*E*→*I*_) via γCaMKII and maintain theta and gamma oscillations, which is essential for the establishment of long-term memory dependent on HPC (He X. et al., 2021). Accumulation of AβO_1–42_ selectively demages the synaptic transmission between CA1 PC and PV + interneurons and disturbs theta-nested gamma oscillations. Meanwhile, AβO_1–42_ interferes with the disinhibition of SST + interneurons on the proximal dendrites of CA1 PC, hence impairing the spike timing-dependent LTP induced by nested gamma oscillation. Optogenetic activation of PV + and SST + interneurons rescues the gamma oscillations and oscillation-induced LTP (Park et al., 2020).

In the brain-wide crosstalk between network oscillations and synaptic plasticity, it is worthwhile to investigate whether enhanced theta and gamma could facilitate the expression of genes related to synaptic connections (He X. et al., 2021). Multiple genes involved in synaptic, intracellular, and vesicle-mediated transport (Syn, vGlut1, etc.) are upregulated by GENUS, and GENUS might be able to modulate the phosphorylation of synaptic proteins (Adaikkan et al., 2019). In the scenario of cerebral ischemia, 40 Hz visual stimulation restores the density of spines in CA1, particularly the mature stubby-shaped spines, while increasing the expression of regulator of G-protein signaling 12 (RGS12) and boosting the LTP of CA3-CA1 synapses via the RGS12-N type voltage-gated calcium channel (N-VGCC) pathway. This reveals that gamma entrainment in the brain can modulate protein expression and synaptic plasticity (Zheng et al., 2020). Clinical researches demonstrated that gamma-band tACS reversed the long-term depression (LTD)-like effect but enhanced the LTP-like plasticity in M1 by inhibiting GABAergic interneurons. Whether gamma tACS regulates synaptic metaplasticity and the activity of cortical GABAergic neurons by a steady or unstable manner remains elusive (Guerra et al., 2018, 2019, 2021, 2022).

### Astrocyte and gamma entrainment

Although neuron assemblies are the terminal apparatus for generating neural oscillations, non-neuronal cells play an important role in supporting energetic metabolism, manipulating synaptic plasticity and preserving microenvironmental homeostasis. Moreover, the notion of glial transmitter system has emphasized the value of glial cells in network oscillations and information processing (Gundersen et al., 2015; Bazargani and Attwell, 2016). Using cholinergic agonists in hippocampal slices, Lee et al. investigated gamma oscillations and found that the transient increase of calcium concentration in astrocytes precedes the onset of oscillations and that the release of astrocyte vesicles is necessary for the maintenance (but not the initiation) of cholinergic-induced gamma oscillations, as well as normal cognition and memory in animals (Lee et al., 2014). Astrocyte specific S100 calcium binding protein B (S100B) in mPFC enhances theta-gamma PAC *in vivo* and improves cognitive flexibility, indicating that astrocytes might participate in the complicated signaling that constitutes the neural circuits of advanced functions (Brockett et al., 2018).

Furthermore, PV + interneurons in mPFC recruit astrocytes to support the generation of gamma oscillations and to rectify decision-making behavior via activating GABA_*B*_R. In mice performing T-maze cognitive tasks, selective depletion of the mPFC astrocytes GABA_*B*_R (GFAP/PFC^Δ*Gb*^) decreases gamma oscillations and impairs decision-making and working memory, whereas optogenetic activation of astrocytes (but not GABAergic interneurons) rescues their cognitive deficits (Mederos et al., 2021). In addition, astrocyte atrophy is observed in various AD model mice. Astrocyte markers glial fibrillary acidic protein (GFAP) and S100B are up-regulated in AC and CA1 of 5xFAD mice treated with GENUS, which might contribute to Aβ clearance and cognition improvement (Martorell et al., 2019).

### Gamma entrainment manipulates microglia

During brain development, microglia modulate synaptic plasticity bidirectionally through spine formation and elimination, while simultaneously serving as dynamic neuron activity monitors (Filipello et al., 2018; Liu et al., 2019; Nguyen et al., 2020; Merlini et al., 2021). In cortical organoids, periodic oscillatory events and NMDA-induced gamma oscillations can be recorded, which is attributable in part to microglia-like cells that support the maturation and differentiation of neurons (Trujillo et al., 2019; Fagerlund et al., 2021). In a mature brain, the interaction between homeostatic microglia and synapses increases neuronal activity and contributes to the synchronized firing of local neuronal populations, which is impaired by LPS-activated microglia (Akiyoshi et al., 2018).

As aforementioned, neuroinflammation and oxidative stress driven by microglia can disrupt normal gamma oscillations (see section “Central nervous system perturbation: inflammation and oxidative stress”). Minocycline, a microglia inhibitor, alleviates neuroinflammation and synaptic loss, which consequently restores gamma oscillations in prefrontal circuit and improves cognitive deficit (Chini et al., 2020; Ji et al., 2020). Since most studies have focused on the destructive outcome of overactivated microglia, the neuroprotection effect of activated microglia is reported recently. LPS-activated microglia replace the inhibitory presynaptic terminals of cortical neurons by synaptic stripping, which increases gamma-band synchrony of cortical neuron firing. Consequently, the increased neuronal activity results in activation of Ca^2+^-mediated CaM kinase IV, phosphorylation of CREB, and a rise in the level of anti-apoptotic and neurotrophic molecules, which contributes to prevent the death of cortical neurons after inflammation stress (Chen et al., 2014). Together, microglia can play either a restorative or destructive role, depending upon the different exposures and relative balance between neuroprotective versus neurotoxic factors.

Microglia play a dual role in the pathophysiology of AD, that is, they can actively uptake and degrade Aβ, but long-term activated microglia tend to produce neurotoxicity and pro-inflammatory substances (Liddelow et al., 2017). GENUS is demonstrated to transform microglia to a phagocytic state with enlarged soma and shortened processes (Iaccarino et al., 2016; Adaikkan et al., 2019; Martorell et al., 2019). Compared to GENUS, acute 40 Hz pulse transcranially delivered focused ultrasound (tFUS) can activate microglia more widely encompassing with Aβ plaque (Bobola et al., 2020). Besides, healthy mice exposed to 40 Hz visual stimulation showed an upregulation of NF-κB and MAPK phosphorylation signaling that is discriminated from the immune response to acute neuroinflammation (Garza et al., 2020). After GENUS, the number of rod-shaped microglia as well as the level of CD40 and C1q are decreased, suggesting a possible anti-inflammatory effect of GENUS (Iaccarino et al., 2016; Adaikkan et al., 2019; Martorell et al., 2019). Our findings revealed the neuroinflammatory mechanism of post-stroke anxiety that HDAC3 is up-regulated in activated microglia in ischemic cortex, which mediates the deacetylation and nuclear translocation of p65 that activates NF-κB pathway and evokes the expression of downstream molecules COX1 and PGE2. PGE2 subsequently interacts with EP2 in amygdala to increase the susceptibility of animals to stress exposure after ischemic stroke. It is noteworthy that we found 40 Hz visual stimulation effective to inhibit the activation of cortical microglia, down-regulate HDAC3/COX1/PGE2/EP2 signaling, and rescue the anxiety-like behavior of animals, indicating that GENUS might be a powerful and non-invasive intervention to manipulate microglia immune response (Zhu et al., 2022). However, GENUS has no significant impact on the number, morphology and immune markers of microglia in elderly C57BL/6J mice (absence of the phenotype observed in P301S and CK-p25 mice). Likewise, few microglial responses are observed in the animal model of ischemic stroke. Thus, whether GENUS is capable to modulate microglia may vary depending upon the disease status or genetic background (Adaikkan et al., 2019; Zheng et al., 2020).

In addition, a number of studies have questioned whether the stimulation frequency 40 Hz, a key metric reported in most researches, is required to manipulate microglia. Visual stimulation at 60 Hz (rather than 40 Hz) increases the expression of CD68 and MMP-9 in microglia in V1 and evokes the degradation of PNNs, representing a non-invasive method to induce the interaction between microglia and PV + neurons and remodel PNN by network oscillation entrainment (Venturino et al., 2021). 1070 nm light stimulation at a pulse frequency of 10 Hz (but not 40 Hz) activates microglia while increasing the co-localization of microglia and Aβ in APP/PS1 mice (Tao et al., 2021). Future studies will be necessary to adequately clarify the mechanisms of oscillatory entrainment operating microglia response, for instance by using single-cell sequencing to determine whether GENUS drives a significant change in microglial gene expression profiles.

## Future remarks

**FIGURE 2:**
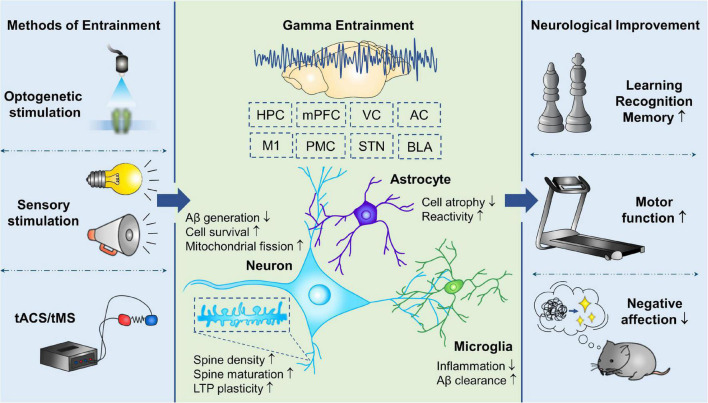
Therapeutic effects of gamma entrainment in CNS diseases. Invasive and non-invasive methods are utilized to evoke gamma entrainment in different brain regions, which provides neuroprotection directly on neurons, as well as modulation of glial reactive states, and consequently improves various neurological functions. tACS, transcranial alternating current stimulation; tMS, transcranial magnetic stimulation; HPC, hippocampus; mPFC, medial prefrontal cortex; VC, visual cortex; AC, auditory cortex; M1, primary motor cortex; PMC, premotor cortex; STN, subthalamic nucleus; BLA, basolateral amygdala; LTP, long-term potentiation.

For several decades, the relationship of gamma oscillations and advanced brain functions has been gradually understood. Gamma entrainment demonstrates therapeutic efficacy in a variety of neuropsychiatric diseases, especially cognitive and memory disorders (Figure 2). Optogenetic technology and even more cutting-edge techniques permit us to customize the activation of brain regions and targeted cells of interest, and investigate the phenomenon of gamma oscillations in either physiological or pathophysiological states, as well as the methods to rescue aberrant gamma rhythm (Sohal, 2012). Cortical organoid provides us a flexible model to observe and manipulate the neural oscillations dynamically at a macroscale and neurotransmitter signals at a microscale during different periods of brain development (Trujillo et al., 2019). Further researches are warranted to validate the phenotype of gamma oscillations in neurological diseases, the mechanism of neuroprotection offered by gamma entrainment and its reliability in clinical treatment. Resolving these outstanding challenges will promote to define gamma entrainment as a non-invasive, cost-effective, and practical therapy, and accelerate its clinical application in the diagnosis and treatment of CNS diseases in the future.

## Author contributions

AG, SW, and AH contributed to search the literature and write the main text. CQ, YL, XL, and JW provided helpful discussions and/or comments in preparing this manuscript. QW and BD critically revised the manuscript. All authors contributed to the article and approved the submitted version.
